# Presumption of guilt for T cells in type 1 diabetes: lead culprits or partners in crime depending on age of onset?

**DOI:** 10.1007/s00125-020-05298-y

**Published:** 2020-10-21

**Authors:** Alexia Carré, Sarah J. Richardson, Etienne Larger, Roberto Mallone

**Affiliations:** 1 Université de Paris, Institut Cochin, CNRS, INSERM, Paris, France; 2 Institute of Biomedical and Clinical Science, University of Exeter Medical School, Exeter, UK; 3 Assistance Publique Hôpitaux de Paris, Hôpitaux Universitaires de Paris Centre-Université de Paris, Cochin Hospital, Service de Diabétologie et Immunologie Clinique, Paris, France

**Keywords:** Autoantibodies, Autoimmunity, Beta cells, Endotypes, HLA, Immunotherapy, Insulitis, Islets, Pancreas, Review, T cells

## Abstract

Available evidence provides arguments both for and against a primary pathogenic role for T cells in human type 1 diabetes. Genetic susceptibility linked to HLA Class II lends strong support. Histopathology documents HLA Class I hyperexpression and islet infiltrates dominated by CD8^+^ T cells. While both hallmarks are near absent in autoantibody-positive donors, the variable insulitis and residual beta cells of recent-onset donors suggests the existence of a younger-onset endotype with more aggressive autoimmunity and an older-onset endotype with more vulnerable beta cells. Functional arguments from ex vivo and in vitro human studies and in vivo ‘humanised’ mouse models are instead neutral or against a T cell role. Clinical support is provided by the appearance of islet autoantibodies before disease onset. The faster C-peptide loss and superior benefits of immunotherapies in individuals with younger-onset type 1 diabetes reinforce the view of age-related endotypes. Clarifying the relative role of T cells will require technical advances in the identification of their target antigens, in their detection and phenotyping in the blood and pancreas, and in the study of the T cell/beta cell crosstalk. Critical steps toward this goal include the understanding of the link with environmental triggers, the description of T cell changes along the natural history of disease, and their relationship with age and the ‘benign’ islet autoimmunity of healthy individuals.

**Electronic supplementary material:**

The online version of this article (10.1007/s00125-020-05298-y) contains a slideset of the figures for download, which is available to authorised users.

**Figure:**
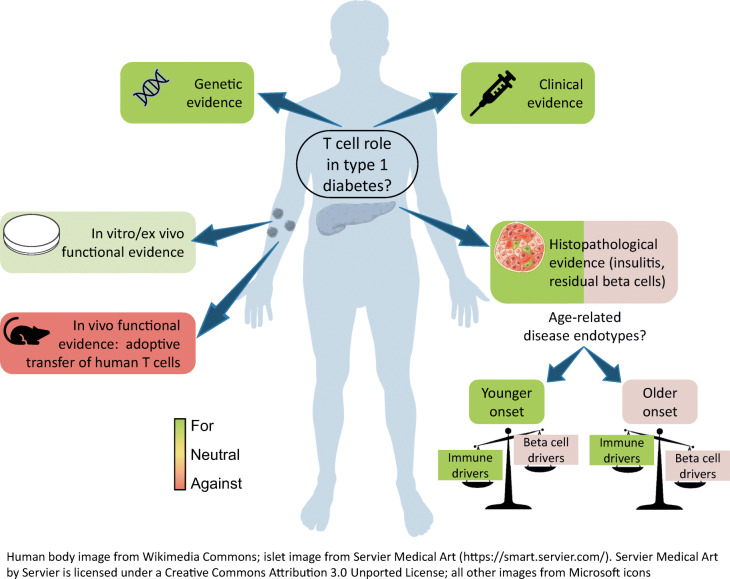
Graphical abstract

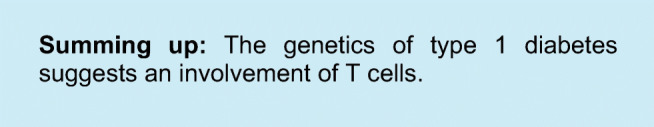

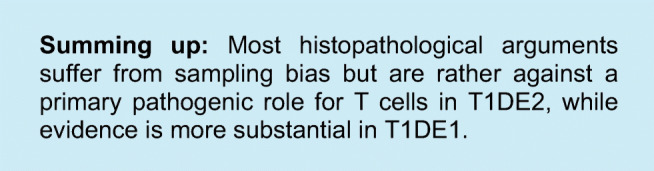

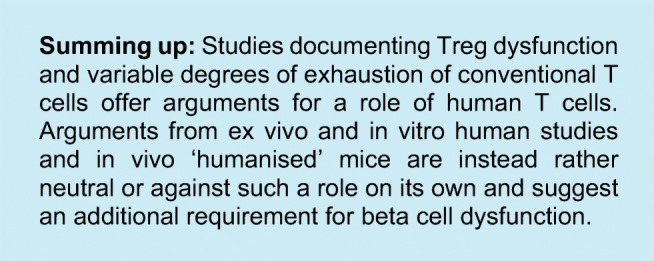

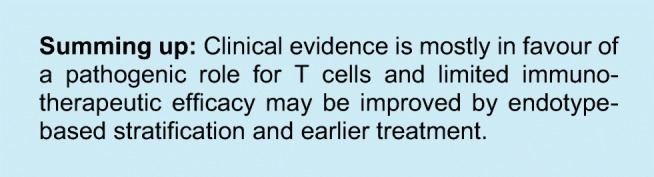

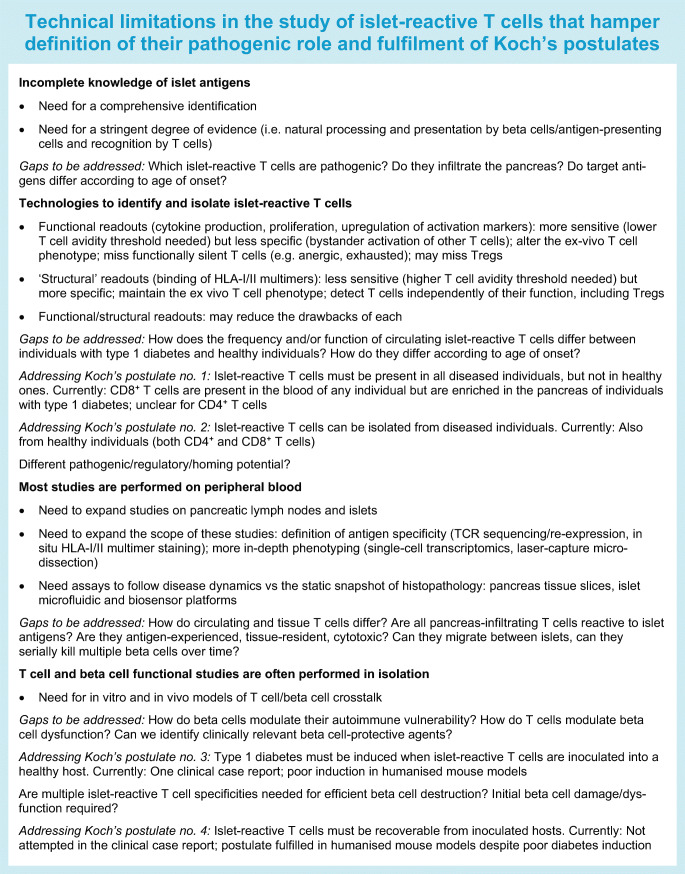











## Introduction

**Fig. 1:**
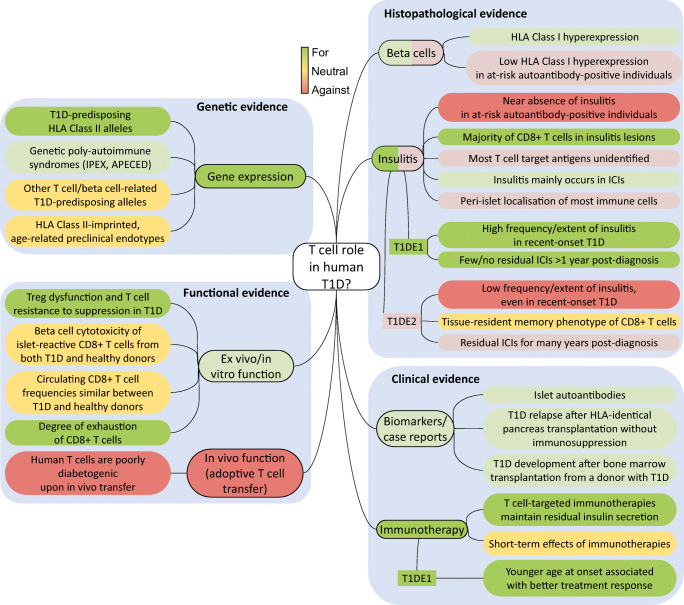
Evidence for or against a primary pathogenic role for T cells in human type 1 diabetes (T1D). APECED, autoimmune polyendocrinopathy, candidiasis, ectodermal dystrophy (syndrome); IPEX, immune dysregulation, polyendocrinopathy, enteropathy, X-linked (syndrome); This figure is available as part of a downloadable slideset

While type 1 diabetes is described as a T cell-mediated autoimmune disease, a more holistic view comprising the dialogue between T cell aggressors and beta cell targets is gaining credit. On one hand, this novel view underlines the active pathogenic role played by beta cells (reviewed in [1]) and on the other, it calls for a critical reappraisal of the role of T cells, which we address here. We contend that evidence for a primary or exclusive role of T cells is variable and probably reflects a balance with mechanisms of beta cell dysfunction that underlie different disease subtypes (endotypes) [2]. These endotypes may thus feature T cells as either ‘lead culprits’ or ‘partners in crime’ for beta cell destruction. We will review arguments for and against T cell-mediated mechanisms (summarised in Fig. 1), link them to age-related endotypes, and propose future directions to settle these questions.

## Genetic evidence

### For

Type 1 diabetes susceptibility and protection are strongly associated with HLA Class II (HLA-II) and, to lesser extent, HLA Class I (HLA-I) loci [3], with HLA-DQB1*06:02 exerting a dominant protection. Given the antigen-presenting function of HLA molecules, these associations support a role for T cells. Several other disease-associated gene variants regulate T cell responses (e.g. *PTPN22*, *IL2RA*, *CTLA4*) [3]. Moreover, disease-associated, and likely causal, genetic variants are enriched in open chromatin (i.e. accessible for transcription) specifically in immune cells, particularly in CD4^+^ effector T cells [4]. The rare genetic poly-autoimmune syndromes immune dysregulation, polyendocrinopathy, enteropathy, X-linked (IPEX) syndrome (*FOXP3* mutations) and autoimmune polyendocrinopathy, candidiasis, ectodermal dystrophy (APECED) syndrome (*AIRE* mutations) affect T cell tolerance mechanisms and can comprise type 1 diabetes. Genetically imprinted, age-related disease endotypes may exist, as younger (<13 months old) children more frequently carry *HLA-DR4/DQ8* and first seroconvert for anti-insulin autoantibodies (IAA), while older children (>40 months old) more often carry *HLA-DR3/DQ2* and present with anti-GAD at first seroconversion [5].

### Against

Although several disease-predisposing alleles are expressed in beta cells and modulate islet inflammation [1], their contribution to disease predisposition is rather small, suggesting a modulatory rather than driving role. Interestingly, HLA-II-imprinted endotypes influence seroconversion [5] but do not strongly affect age of onset [6], clinical progression or C-peptide loss post-diagnosis [7], indicating that other genetic and/or acquired factors determine the progression rate of beta cell damage.

## Histopathological evidence

### For

HLA-I hyperexpression on beta cells [8, 9] suggests a role for CD8^+^ T cells. The second histopathological hallmark is the presence of immune infiltrates (insulitis) dominated by CD8^+^ T cells, followed by CD68^+^ macrophages, CD20^+^ B cells and CD4^+^ T cells [10–13]. Scattered neutrophils are also observed throughout the pancreas [14, 15] and evidence of dysregulated systemic innate immunity has been consistently reported, even before autoantibody seroconversion [14]. The relative under-representation of CD4^+^ T cells and the near absence of regulatory T cells (Tregs) may reflect an earlier, more peripheral role (e.g. in pancreatic lymph nodes), while the over-representation of CD8^+^ T cells may reflect a final effector role in beta cell destruction.

**Fig. 2:**
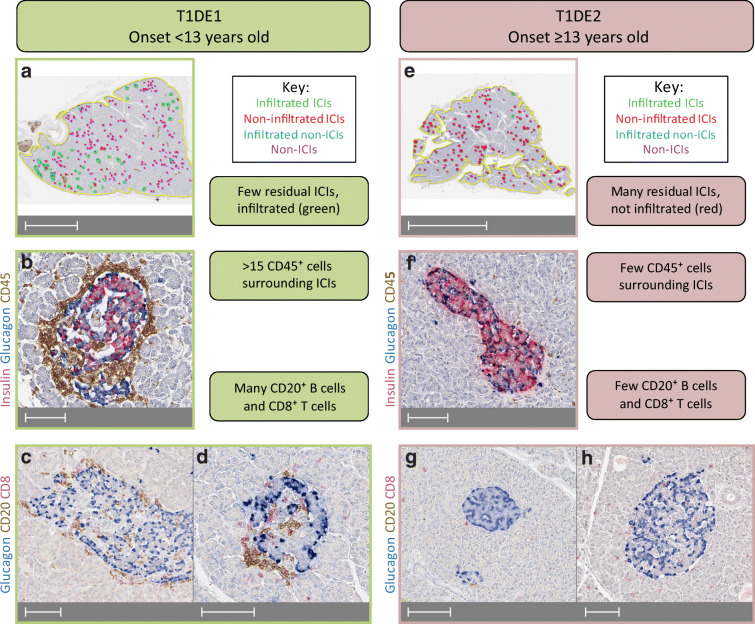
Key differences in insulitis and residual ICIs in type 1 diabetes endotypes T1DE1 and T1DE2. (**a**–**d**) T1DE1 donors (mostly with a disease onset <13 years old) have few residual ICIs (**a**; green and red circles) compared with non-ICIs (blue and pink circles). The majority of residual ICIs display significant immune infiltration (**a**; >15 CD45^+^ cells, green circles); a representative image is shown in (**b**). This infiltrate is enriched in CD20^+^ B cells and CD8^+^ T cells (**c**, **d**). (**e**–**h**) T1DE2 donors (mostly with a disease onset ≥13 years old) retain significant residual ICIs (**e**; red and green circles), the majority of which do not meet the criteria for insulitis (**e**; red circles). Most ICIs have limited infiltration with CD45^+^ cells (**f**), the majority of which are CD8^+^ T cells with few CD20^+^ B cells (**g**, **h**). Specimens from representative donors from the EADB biobank (https://foulis.vub.ac.be) were immunostained with antibodies against insulin/glucagon/CD45 or glucagon/CD20/CD8 using triple chromogen-based immunohistochemistry similar to that described in [78]. Scale bars, 5 mm (**a**), 100 μm (**b**–**d**, **f**–**h**) or 10 mm (**e**). This figure is available as part of a downloadable slideset

Studies on the Exeter Archival Diabetes Biobank (EADB) documented that insulitis of donors with recent-onset type 1 diabetes differs according to age [13, 16, 17], suggesting the existence of two endotypes with different pathogenic mechanisms (Fig. 2): type 1 diabetes endotype 1 (T1DE1), in individuals with onset at age <13 years; and type 1 diabetes endotype 2 (T1DE2), mostly occurring in individuals diagnosed at ≥13 years of age. T1DE1 is characterised by a CD20^high^ insulitis rich in CD8^+^ T cells, few residual insulin-containing islets (ICIs; indicative of residual beta cells) and evidence of abnormal insulin processing in the remaining beta cells. In contrast, T1DE2 displays a CD20^low^ insulitis with fewer CD8^+^ T cells, more residual ICIs and normal insulin processing. Recent-onset (<6 month) CD20^high^ donors have a median of 78% of insulitis-positive ICIs (range 19–100%; *n* = 20), vs 14% (range 0–52%; *n* = 19) in CD20^low^ donors (SJR, unpublished data). In line with this endotype view, the frequency of insulitis within 1 month of diagnosis is 73% in young donors with type 1 diabetes (<14 years old, likely to be mostly T1DE1) [18] and the parallel reduction in residual ICIs may indicate an aggressive beta cell destruction. In contrast, insulitis within 1 month from diagnosis is less frequent (29%) in older donors (15–40 years old, likely T1DE2) and long-term ICI preservation suggests reduced aggressiveness [19].

### Against

If viewing type 1 diabetes as a single disease, the histopathological evidence for a critical T cell involvement is unconvincing. Immune infiltrates are limited, as exemplified by the debate on the diagnostic definition of insulitis being the presence of ≥15 immune (CD45^+^) cells/islet in ≥3 islets [20, 21]. This definition should be interpreted by considering the following points: (1) 15 immune cells/islet is only twice the number found in non-diabetic donors [10]; (2) it does not account for differences in islet size (i.e. the insulitis density); (3) infiltrated islets are typically few (<10%) and found in a minority (~20%) of donors [18, 22]; (4) peri-islet, non-invasive insulitis is more common [19]; and (5) lymphocytes are also found scattered in the exocrine tissue, even in autoantibody-positive non-diabetic donors [12]. Moreover, insulitis is mostly confined to ICIs (33% vs 2% in non-ICIs) and beta cell area and mass are higher in donors with type 1 diabetes with insulitis than in those without insulitis [22]. These patterns may indicate that immune cells leave the islets once beta cell destruction is complete.

These findings become more convincing when interpreted according to age-related endotype differences. However, a major bias is introduced by the specimens available, which are very limited for children with recent-onset type 1 diabetes because, fortunately, very few still die close to disease onset. For instance, the Network of Pancreatic Organ Donors with Diabetes (nPOD) has collected only one such specimen since 2007. In contrast, most tissues are available for T1DE2 donors with older-onset type 1 diabetes (i.e. those with weaker evidence for a major T cell role). In most studies, age-related endotype differences are further blurred by the analysis of specimens from individuals with overall long disease durations, which, together with age, influence the extent of insulitis and the number of residual ICIs. For instance, the frequency of insulitis in young donors with type 1 diabetes (<14 years old) drops from 73% within 1 month of diagnosis to 4% beyond 1 year [18]. Thus, even for the younger histopathological T1DE1 with stronger evidence for a major T cell role, key hallmarks have likely waned in most long-standing diabetes specimens available. Together with the focus of most studies on older-onset donors, these limitations may bias our view against T cells.

The bias of long disease duration is absent in the Diabetes Virus Detection Study (DiViD), which collected surgical pancreas specimens from living adults with new-onset disease [23]. In these donors, 11% of the islets displayed insulitis, a proportion comparable with that observed in similar age/duration-matched CD20^low^ donors in both EADB and nPOD specimens. The fact that these samples came from living donors rules out perimortem or post-mortem changes as possible confounders. Collectively, the DiViD, EADB and nPOD findings with older-onset but short-duration disease rule out the possibility that the limited insulitis observed represents an extinguished phase of islet autoimmunity, as could be the case in donors with longer disease duration.

Even in individuals with recent-onset disease, the autoimmune process dates back several months or years and the situation could be different at the preclinical disease stages: stage 1, presence of ≥2 autoantibodies; stage 2, presence of autoantibodies and initial metabolic alterations (e.g. loss of first-phase insulin secretion); and stage 3, presence of autoantibodies and overt hyperglycaemia (i.e. clinical disease) [24]. One single autoantibody marks a low risk of progression, especially in adults, and is therefore not included in this staging system. Yet, most autoantibody-positive pancreas donors analysed to date are adults who are positive for a single autoantibody. It should be noted, however, that the low-risk definition of the single autoantibody status is based on longitudinal assessments in living humans and that the probability of appearance of a second autoantibody is approximately 7–20% at 5 years (depending on age), with a subsequent risk of clinical progression similar to those individuals who are positive for multiple autoantibodies from the start [25]. Thus, out of the reasonably large series of ~100 donors positive for a single autoantibody analysed to date, a fraction would have eventually progressed. Yet, insulitis was invariably absent in all autoantibody-positive donors, even in those with predisposing HLA haplotypes [19]. Additionally, in the relatively limited number of specimens from donors positive for multiple autoantibodies (i.e. stage 1 disease; ~20 analysed to date), little or no insulitis was observed (1–9% of islets) [10, 22, 26]. This picture may reflect a disease kinetics remaining stable for many years before sudden progression. Arguably, snap-shooting this ‘point of no-return’ may be difficult in histopathological studies.

HLA-I hyperexpression is also typically found in ICIs; expression decreases with disease duration and is often associated with CD8^+^ insulitis [27]. However, it is more readily apparent than insulitis and is present in most ICIs, probably reflecting a readout of beta cell stress. A possible interpretation of this discrepancy is that insulitis hallmarks may be more labile due to immune cells migrating between islets and the peri-islet and exocrine tissue, resulting in a variable pattern at any given time point compared with the more stable HLA-I hyperexpression. Nonetheless, even HLA-I hyperexpression was only found in 13% of islets from individuals positive for two autoantibodies, associated with higher yet mild CD8^+^ T cell infiltration [28].

Another point of controversy is that, despite the identification of several antigen specificities among pancreas-infiltrating T cells, a large fraction remains unassigned. Moreover, whether these T cells present an effector/memory phenotype compatible with an active autoimmune engagement is unknown. One study suggested a prevalent (46%) tissue-resident memory phenotype of peri-islet CD8^+^ T cells [29] that is enriched in non-diabetic donors [30] and, possibly, individuals who are T1DE2 [16, 29]. Although described as non-cytotoxic in insulitis lesions [29], tissue-resident T cells can proliferate locally, produce proinflammatory cytokines and recruit circulating cells, thus possibly contributing to perpetuate disease. Mouse studies [31, 32] suggest that a significant fraction of pancreas-infiltrating T cells are naive (also in humans [15]) and non-islet-reactive, possibly configuring insulitis as an ‘open’ lesion in which bystander T cell activation may contribute to beta cell damage.

## Functional evidence

### For

Although the NOD mouse model is imperfect, both CD4^+^ and CD8^+^ T cells are needed to transfer disease [33]. Moreover, NOD mice lacking MHC Class I (MHC-I) do not develop diabetes unless receiving splenocytes from diabetic animals [34]. Interestingly, beta cell-selective MHC-I knockout in mice [35] protects against diabetes but not insulitis, suggesting that MHC-I-interacting CD8^+^ T cells exert a late pathogenic role, consistent with their final involvement in beta cell cytotoxicity. The antigen specificity of these T cells has been explored with T cell receptor (TCR)-transgenic NOD mouse studies, which suggest a variable pathogenic potency of different T cell clonotypes. Indeed, diabetes development is accelerated by some TCRs, such as the CD4^+^ TCRs BDC-6.9 [36] (recognising a proinsulin/islet amyloid polypeptide hybrid peptide [37]) and 4.1 of unknown specificity [38] and the CD8^+^ islet-specific glucose-6-phosphatase catalytic-subunit related protein (IGRP)_206–214_-reactive 8.3 TCR [38]). Others do not accelerate diabetes development (i.e. the CD4^+^ TCR BDC-2.5 reactive to a proinsulin/chromogranin-A hybrid peptide [37]), unless on a NOD/*scid* immunodeficient background [39], and the CD8^+^ Ins_B15-23_-reactive G9C8 TCR, which requires prior Ins_B15-23_ immunisation [40]).

Reports analysing Tregs concluded that, while the frequency of circulating forkhead box P3 (FOXP3)^+^CD4^+^ Tregs is unaltered in type 1 diabetes, their regulatory activity is diminished [41]. This alteration reflects both a reduced suppressive function of Tregs [42], sometimes associated with increased secretion of proinflammatory cytokines (IFN-γ, IL-17) [41, 43], and an increased resistance of conventional T cells to suppression [44]. Treg dysfunction is partly genetically imprinted (e.g. through *IL2RA* polymorphisms leading to unstable FOXP3 expression under limiting IL-2 concentrations [41]). It is also highly heterogeneous across individuals and largely overlapping with non-diabetic control individuals, possibly pointing to Treg-driven disease endotypes. Similarly, IL-10-polarised islet-reactive CD4^+^ T cells were enriched in healthy donors and individuals with type 1 diabetes of later onset [45], who may be representative of T1DE2 [13].

### Against

Animal models of autoimmune diseases were initially produced by immunisation with organ extracts or antigens (e.g. for multiple sclerosis (1933) [46], orchitis, thyroiditis [47], adrenalitis, rheumatoid arthritis [48]). Despite a better knowledge of target antigens, similar immunisations of non-transgenic animals never provided an equivalent model of experimental insulitis/diabetes, possibly reflecting the requirement for beta cell dysfunction. The BioBreeding rat provides another spontaneous animal model of human type 1 diabetes. Interestingly, it harbours a profound systemic T cell lymphopenia, including near absence of CD8^+^ T cells, which, despite some CD8^+^ T cell infiltration in islets [49], is required for spontaneous diabetes development [50].

CD8^+^ T cell clones recognising preproinsulin [51], IGRP [52] and zinc transporter 8 [53] epitopes can lyse beta cells in vitro. However, we documented that T cell clones from healthy donors display similar cytotoxic potency [53]. Although this similarity could reflect the long-term in vitro stimulation of these clones, erasing ex vivo differences, it demonstrates that islet-reactive CD8^+^ T cells from any individual can be differentiated into cytotoxic effectors. Albeit this does not negate a pathogenic role for T cells, it does not provide supportive evidence either, as frequently claimed. Moreover, most in vitro cytotoxic experiments [51, 52] employed high effector/target ratios (10/1 to 25/1) compared with those observed in situ in the pancreas [8].

These results mirror recent observations by us [53–55] and others [56–58] that islet-reactive CD8^+^ T cells circulate at similar frequencies in healthy individuals and individuals with type 1 diabetes. It is unclear whether the same applies to CD4^+^ T cells. These reports detected CD8^+^ T cells using HLA-I multimers (i.e. independent of their functional phenotype), while a functional ELISpot readout of IFN-γ secretion repeatedly documented disease specificity [51, 53, 59, 60]. Thus, islet-reactive CD8^+^ T cells may differ according to disease status in terms of functional profile (e.g. exhaustion) rather than frequency [57, 61] and in their capacity to home to the pancreas, where their density is enriched in donors with type 1 diabetes [53, 54]. This difference in homing may point to the heightened vulnerability of stressed/senescent beta cells as an important driver [1, 62, 63]. Overall, these results open key questions to better understand T cell autoimmunity and biomarkers but do not offer arguments either for or against a primary pathogenic role for T cells.

Human T cell adoptive transfer into immunodeficient mice was not diabetogenic (e.g. using an IGRP-reactive CD8^+^ T cell clone [52]). Although TCR-transgenic CD4^+^ T cells recognising the HLA-DQ8-restricted INS_B9-23_ epitope transferred into HLA-DQ8-transgenic mice reconstituted with human thymus and CD34^+^ cells induced diabetes [64], they required strong priming conditions (i.e. multiple low-dose streptozotocin and INS_B9-23_ peptide immunisation). Streptozotocin-induced beta cell death and antigen release may provide the beta cell dysfunction signal needed for efficient T cell priming.

## Clinical evidence

### For

Islet autoantibodies appear before disease onset [5] and are produced by B cells, thus implying a helper role provided by CD4^+^ T cells. Eight case reports documented disease relapse in non- or minimally immunosuppressed recipients with type 1 diabetes 6–12 weeks after pancreas transplantation from non-diabetic HLA-identical siblings, with no relapse observed under immunosuppression [65]. Conversely, a case report described disease development 4 years after non-T cell-depleted HLA-identical bone marrow transplantation from a donor with type 1 diabetes [66].

Among the panoply of immunotherapeutic agents trialled in individuals with new-onset type 1 diabetes, four have shown some effect on C-peptide preservation: LFA-3-Ig (alefacept) [67], CTLA-4-Ig (abatacept) [68], anti-CD20 (rituximab) [69] and anti-CD3 monoclonal antibodies [70–73]. Apart from the anti-B cell agent rituximab, all target mainly T cells, arguing for T cell-driven mechanisms targetable by drugs. Interestingly, superior clinical benefit is often observed in children [67, 69, 70, 72], who may represent the more T cell/B cell-driven endotype T1DE1. Concordantly, individuals diagnosed at younger ages generally lose C-peptide secretion more rapidly and the fast-progressing subgroup displays age-dependent blood gene expression and cell count profiles that are higher for B cells and lower for neutrophils [7]. Moreover, individuals with higher pre-treatment B cell counts achieve superior C-peptide preservation after rituximab treatment [7], exemplifying the relevance of disease endotypes to selection of more-targeted immunotherapies.

### Against

The limited and partial improvements observed in immunotherapy trials starkly contrast with the more significant benefits achieved in other autoimmune diseases. Examples of mainstay disease-modifying therapies include the following: IFN-β, glatiramer, dimethyl fumarate, sphingosine-1-phosphate receptor modulators and antibodies to very late antigen-4 and CD20 for multiple sclerosis [74]; and methotrexate, TNF and IL-6 inhibitors, rituximab and abatacept for rheumatoid arthritis [75]. However, all these agents perform much better when given early in the disease course, at a stage that might correspond to the preclinical phase of type 1 diabetes.

A recurrent observation from immunotherapy trials in individuals with new-onset diabetes is that the effect on C-peptide preservation is limited to the first months of treatment and resumes its decline thereafter [76]. While this may indicate a need for prolonged treatment, it could also reflect targeting of only the T cell pathogenic component, leaving others, possibly related to beta cells [1], free to drive further disease progression. However, trials with putative beta cell-protective agents (e.g. glucagon-like peptide-1 agonists) have so far failed to deliver significant clinical benefits, or to convincingly ascribe any such benefit to beta cell protection [77].

## Gaps in knowledge preventing conclusive evidence of the pathogenic role of T cells

The fulfilment of Koch’s postulates for a pathogenic role for T cells is currently incomplete, partly due to technical limitations hindering progress (see Text box: ‘Technical limitations in the study of islet-reactive T cells that hamper definition of their pathogenic role and fulfilment of Koch’s postulates’). Conceptually, our understanding remains limited at two levels. First, it is not clear how environmental factors trigger T cell engagement. Most candidates (e.g. enteroviral infections and nutrients disrupting the gut barrier and/or microbiota composition) could exert their effects on both T cells and beta cells. Thus, the relative weight and temporal sequence of these effects would clarify whether T cell engagement represents a primary causative event or a bystander consequence of beta cell dysfunction, and whether such engagement affects disease initiation and/or progression/amplification. We also need to better understand the intermediate steps (e.g. metabolic and inflammatory derangements) connecting environmental triggers with T cell recruitment, and the innate-adaptive immune crosstalk. Second, differences in the frequency and/or phenotype of circulating islet-reactive T cells between diabetic and healthy donors are rather subtle if not absent altogether. We need to better understand the features of this universal state of ‘benign’ autoimmunity, how it is lost and its relationship with age. To this end, a detailed description of T cell modifications along the natural history of disease is required but remains largely unexplored in peripheral blood and even more so in the pancreas. Very few organ donors analysed are positive for multiple autoantibodies (stage 1 disease), and none are representative of stage 2 with initial dysglycaemia. We can speculate on whether insulitis may represent a transient phase in disease progression. Its near absence in stage 1 donors may thus reflect a relatively late autoimmune acceleration during stage 2 that we are presently missing.

## Conclusion

Figure 1 summarises the available evidence for or against a primary pathogenic role for T cells in type 1 diabetes. While most of the evidence favours such a role, some does not provide the support often given as granted. Histopathological evidence yields a more conflicting picture. Although biased by some limitations inherent to these studies, they suggest the existence of two age-related endotypes. Clinical evidence of faster beta cell loss and superior benefit of immunotherapeutic intervention in individuals with younger-onset diabetes further support this endotype view. Several technical limitations and gaps in knowledge need to be filled to gauge T cell involvement according to age and disease endotypes. Type 1 diabetes may be a case of one name but two diseases (i.e. younger-onset with primary T cell-driven mechanisms and older-onset with primary beta cell-driven mechanisms) leading to similar clinical presentations but requiring different treatments.

## Electronic supplementary material

ESM (PPTX 1.35 mb)

## Abbreviations

DiViD: Diabetes Virus Detection Study
EADB: Exeter Archival Diabetes Biobank
FOXP3: Forkhead box P3
HLA-I: HLA Class I
HLA-II: HLA Class II
ICI: Insulin-containing islet
IGRP: Islet-specific glucose-6-phosphatase catalytic-subunit related protein
MHC-I: MHC Class I
nPOD: Network for Pancreatic Organ Donors with Diabetes
TCR: T cell receptor
T1DE1: Type 1 diabetes endotype 1
T1DE2: Type 1 diabetes endotype 2
Treg: Regulatory T cell
