# Supplementary material for: Presumption of guilt for T cells in type 1 diabetes: lead culprits or partners in crime depending on age of onset?
Source: Diabetologia. 2020 Oct 21;64(1):15–25. doi: 10.1007/s00125-020-05298-y (PMC7717061; doi:10.1007/s00125-020-05298-y)
Supplement: Supplementary file 1 — (PPTX 1.35 mb) [file 125_2020_5298_MOESM1_ESM.pptx]

## Slide 1
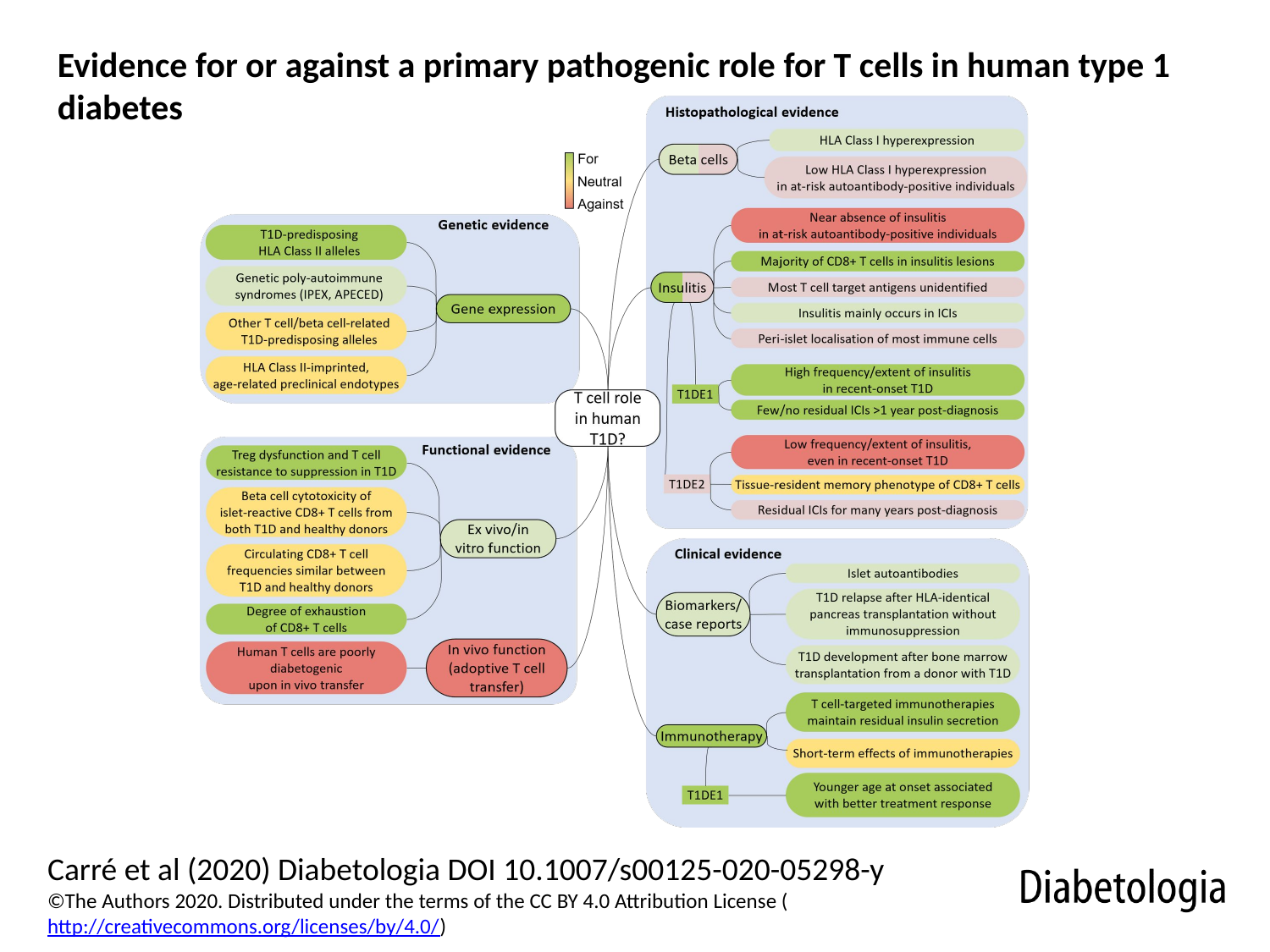

Evidence for or against a primary pathogenic role for T cells in human type 1 diabetes
Carré et al (2020) Diabetologia DOI 10.1007/s00125-020-05298-y
©The Authors 2020. Distributed under the terms of the CC BY 4.0 Attribution License (http://creativecommons.org/licenses/by/4.0/)

## Slide 2
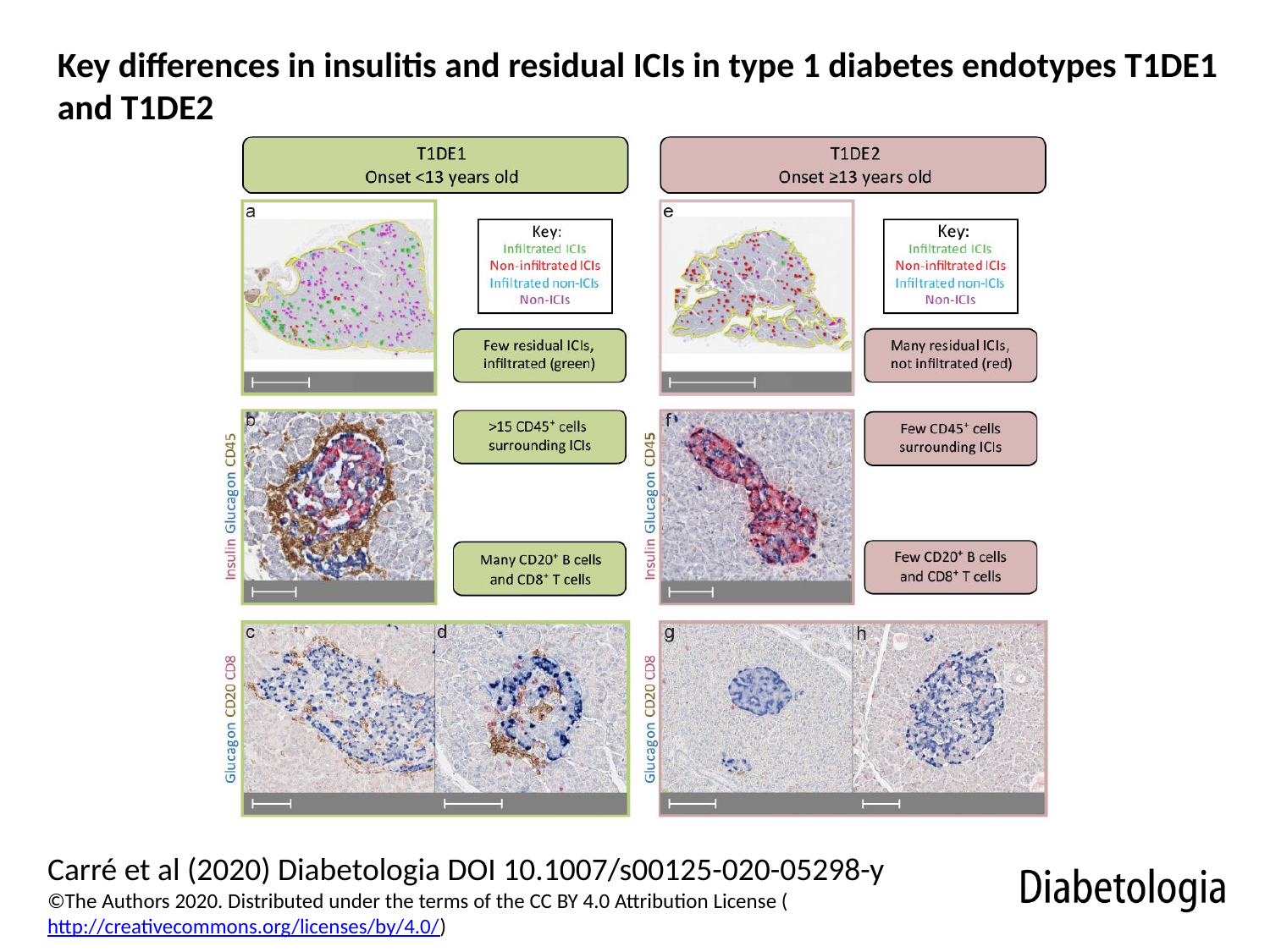

Key differences in insulitis and residual ICIs in type 1 diabetes endotypes T1DE1 and T1DE2
Carré et al (2020) Diabetologia DOI 10.1007/s00125-020-05298-y
©The Authors 2020. Distributed under the terms of the CC BY 4.0 Attribution License (http://creativecommons.org/licenses/by/4.0/)
